# Primary Care Providers’ Perspectives of Knowledge and Skill Acquisition, and Benefits Following University Faculty-Led Continuing Medical Education Sessions: Results of a Qualitative Assessment in Southwest Uganda

**DOI:** 10.7759/cureus.102045

**Published:** 2026-01-21

**Authors:** Edgar Mulogo, Moses Ntaro, Eleanor Turyakira, Michael Matte, Andrew Wesuta, Fred Mwebembezi, Femus Agaba, Prudence Nabimanya, Angela Tushabe, Peter Kawungezi

**Affiliations:** 1 Department of Community Health, Mbarara University of Science and Technology, Mbarara, UGA

**Keywords:** cme, knowledge and skills acquisition, perspectives, primary care provider, south-west, uganda, university led

## Abstract

Background and objective

Mbarara University of Science and Technology’s (MUST) First Mile Community Health Program (FMCH) has been at the forefront of providing continuing medical education (CME) to primary care providers working in health facilities in southwestern Uganda. However, little is known about how CME has influenced primary care providers' knowledge and skills. This qualitative assessment sought to describe participants' perspectives on knowledge and skill acquisition following these CME sessions.

Methods

In 2024, a descriptive review was carried out using activity reports from CME sessions. The review included CME sessions conducted over two years (2018-2019). Forty primary care providers from 40 health facilities, ranging from health centers (HCs) to general hospitals, participated in each CME session offered by MUST faculty. CME sessions were provided in the following areas: basic emergency obstetric care (BEmOC), emergency triage, assessment, and treatment, admission care for the sick child and newborn (ETAT+), and infection prevention and control.

Results

Primary care providers reported gains in knowledge and skills as well as other benefits after attending CME sessions, particularly in newborn care, the manual vacuum aspiration (MVA) procedure, management of pre-eclampsia, birth monitoring, and infection prevention and control. The average pre-test score was 58%, while the average post-test score was 80%. The percentage change in knowledge was 22%. The providers reported incorporating previously overlooked skills or procedures into their clinical practice.

Conclusions

University-led CME sessions can be an effective method for primary care providers working in resource-limited settings to acquire knowledge and enhance their skills. Fostering partnerships between universities and stakeholders in the local health system to support the sustainability of CMEs is likely to have a positive impact on healthcare delivery.

## Introduction

It has been increasingly acknowledged that strengthening the health system is only possible if there are sufficient human resources for healthcare who possess the competence to deliver the care that patients and populations need [1]. Among the educational approaches that enhance knowledge and skills in the medical field, continuing medical education (CME) is the most significant [2]. CME can be described as "any activity that is intended to maintain, develop, or increase the knowledge, skills, professional performance, and relationships that a physician uses to provide services to patients, the public, or the profession" [1,3-5].

The advancement of medical practices, continuous updates in medical knowledge, and ongoing policy changes in the healthcare system mean that healthcare workers face significant learning challenges [6]. This implies that a medical doctor, dentist, pharmacist, nurse, clinical officer, or laboratory technologist may no longer be current in the specialized knowledge and skills acquired during their education, and may therefore become professionally and functionally outdated [7]. Furthermore, at the time of graduation, no practitioner possesses all the knowledge and skills required for professional practice [8]. As a result, updating and restoring these skills is necessary to provide effective healthcare [7,9].

Medical workers can receive CME through various channels, including workshops, seminars, formal lectures, radio and television programs, and supervisory support [9]. In some countries, medical universities are responsible for the health of the population in a specific area, including training care providers in disease prevention [10]. An underlying assumption is that CME can enhance healthcare practice and, as a result, improve patient health outcomes. Educational meetings vary widely in terms of content, number of participants, level and type of interaction, duration, frequency, and targeted practices [11]. These factors can therefore influence the effect of CME on professional practice.

Primary care in Uganda is delivered through the district health system, which includes Health Center I (Village Health Teams), Health Center IV (HC IV), and General Hospitals (GH). Medical doctors are posted to public primary care facilities at the HC IV and GH levels, where they provide first-contact promotional, preventive, curative, rehabilitative, and palliative care [9]. Mbarara University of Science and Technology’s (MUST) First Mile Community Health Program (FMCH) has played a leading role in providing CME to primary care providers in health facilities across southwestern Uganda. CME is essential for primary care providers to maintain, enhance, and update their professional competence [12].

In Uganda, CME offers an opportunity to earn credits for continuing professional development. This suggests that, in some cases, care providers attending CME sessions may be more focused on earning credits than on acquiring new skills. There is limited data in the country on primary care providers' perceptions of the impact of CMEs. However, CME has gained increased attention due to the direct links between clinicians' knowledge, performance in the field, and patient outcomes [13]. The goal of this study is to explore primary care providers' perspectives on the impact of CME provided by MUST. To ensure that accredited CME sessions are valued, it is important to consider the perspectives of multiple stakeholders [1].

## Materials and methods

Study design and setting

A descriptive review using activity reports from CME sessions at the primary care levels of the Ugandan health system was conducted in 2024. The review covered a two-year period during which the CME sessions were held (2018-2019). The primary care levels of service in Uganda are detailed in Table 1.

**Table 1:** Levels of primary care in Uganda

Level	Healthcare services provided	Management of the facility
Village Health Team (VHT)	Community-based preventive and promotive health services, surveillance/screening for diseases, referral, and treatment support. No physical infrastructure	Village Health Team coordinator
Health Centre (HC) Level II	In addition to VHT level services, the HC Level II provides preventive and promotive, outpatient, curative, and emergency delivery	Registered nurses with diploma-level training
Health Centre Level III	In addition to HC Level II services, the HC Level III provides preventive and promotive, outpatient, curative, maternity, inpatient, and laboratory services	Clinical officers with diploma training
Health Centre Level IV	In addition to HC level III services, the HC Level IV provides obstetric, ultrasound, emergency/simple surgery (cesarean sections and lifesaving operations), blood transfusion services, and mortuary	Medical officers with bachelor's degrees
General Hospital	In addition to HC Level IV services, General Hospitals provide services for general medical and surgical conditions, specialized services in Medicine, Surgery, Pediatrics, Community Medicine, Obstetrics and Gynecology, Laboratory and Radiology, Community/Public Health department. It also provides in-service training and basic research	Specialists or medical officers

The primary care providers who attended the CMEs were recruited from health facilities (HC III, HC IV, and General Hospitals) across 19 districts in southwestern Uganda. Mbarara University of Science and Technology’s FMCH program has played a leading role in providing CME to primary care providers in health facilities in this region. The FMCH program's goal is to promote the acquisition of knowledge and skills among primary care providers, thereby enhancing service delivery and ultimately improving regional health outcomes.

The FMCH used a workshop-based approach to conduct the CME sessions. Every quarter, primary care providers were invited to MUST, where sessions were held for individuals from 40 health facilities at a time. The sessions were conducted by faculty from MUST's clinical departments, who led the sessions. The clinical disciplines included basic emergency obstetric care (BEmOC), comprehensive emergency obstetric care (CEmOC), emergency triage assessment and treatment, admission care for sick children and newborns (ETAT+), and infection prevention and control.

In August 2018, 35 of 40 leaders (88%) from primary care level health facilities participated in a training needs analysis, which guided the selection of topics covered in the CMEs. The care providers were divided into five groups and asked to prioritize the listed training needs. Conducting a training needs analysis is essential for developing effective CME courses. By identifying the actual training needs of primary care providers, CME programs can be designed to address specific gaps in knowledge and skill development [11]. A similar approach to identifying training needs has been used in other settings [4]. The identified training needs are presented in Table 2.

**Table 2:** Perceived training needs of the healthcare providers IMNCI: Integrated Management of Newborn and Childhood Illnesses; HIV: human immunodeficiency virus; TB: tuberculosis

Group 1	Group 2	Group 3	Group 4	Group 5
Rational drug use	IMNCI	Integrated management of childhood illness	Malnutrition management	Data management
Emergence obstetric care	Basic obstetric care	HIV care/management	Customer care	Emergency care
Neonatal care	Infection control	Community and facility-based DOTS for TB patients	TB case detection nursing, other staff	Maternal and newborn care
Integrated management of severe acute malnutrition	Data management	Management of obstetrics and gynecological care	Date review	Skilling community resource personnel
Infection prevention and control	Nutrition	Data management (documentation and analysis, computer skills)	Infection control/patient safety	Leadership management and governance
Healthcare waste management	Continous quality improvement + 5s	Emergency preparedness and management (animal bites, poisoning/drug abuse, trauma)	Leadership and human resource management	Customer care
TB/HIV management	Customer-based health intervention	Post abortion care		Health education and promotion
Community linkage framework	Prevention of mother-to-child transmission of HIV	Infection control (waste segregation)		
Management of non-communicable diseases	Rational use of medicine	Rational drug use and dispensing		
	Comprehensive emergency obstetric care	Patient and health worker rights		
	Emergency medical care	Ethical code of conduct		
	Performance management	Maternal, child, and neonatal healthcare		
	Financial management			

Data collection and sample

Following each CME session for primary care providers, an activity report was produced, and a manual deductive thematic analysis was conducted. The reports included information on pre- and post-test scores, topics covered, and participants’ qualitative feedback on the CME sessions. The findings of this study were based on the perceptions of 35 primary care providers who attended a CME session. The analysis identified themes related to perceived knowledge and skill acquisition, as well as the benefits of the CME approach for primary care service delivery. The findings are presented according to the identified themes, with relevant quotes attributed to primary care providers.

## Results

The primary care providers were drawn from different levels of healthcare facilities, as shown in Table 1. Health services begin at the community level through Village Health Teams, which provide preventive care, health education, disease surveillance, and basic treatment directly to individuals, while referring more complex cases to health facilities. HC II introduces outpatient services, basic treatment, and emergency deliveries, overseen by qualified nurses skilled in addressing common health conditions. HC III builds on this by providing inpatient care, maternity services, and basic laboratory diagnostics, supervised by clinical officers who link community care with hospitals. HC IV addresses more complex health needs by performing surgeries such as cesarean sections, offering ultrasound services and blood transfusions, and managing emergencies, all under the oversight of medically trained officers with advanced qualifications. General hospitals then offer comprehensive care across various specialties, including pediatrics, surgery, obstetrics, and internal medicine, while also supporting training and research activities. 

The primary care providers expressed several training needs, as shown in Table 2. Providers across the health system expressed a strong desire to strengthen their skills in both clinical care and health system management. They identified a need for training in maternal, neonatal, and child health, including emergency obstetric care, neonatal care, malnutrition management, and HIV and TB services, reflecting the frontline challenges they face daily. At the same time, there was a clear need for training in infection prevention, patient safety, and rational drug use, highlighting the importance of delivering quality and safe care.

The providers also recognized the growing role of data and information management, seeking training in documentation, analysis, and data-driven decision-making. Beyond clinical and technical skills, there was a call for capacity development in leadership, governance, customer care, community engagement, and ethical practice, demonstrating that effective healthcare relies on strong management and human-centered approaches. Together, these expressed needs reflect a workforce committed to delivering better patient outcomes, improving system efficiency, and building stronger connections with the communities they serve. The focus extends from direct patient care to leadership and data-driven decision-making, illustrating a holistic vision for professional growth and health system strengthening. The profile of the primary care providers who participated in the CME is presented in Table 3. The pre-test/post-test scores for the primary care providers are shown in Table 4.

**Table 3:** Profile of primary care providers SD: standard deviation

Characteristic	Values (N=35)
Age, years, mean, (SD)	37, (9.48)
Gender, n (%)	
Male	24 (68.57%)
Female	11 (31.43%)
Level of training, n (%)	
Medical officer	18 (51.43%)
Clinical officer	13 (37.14%)
Nurse	4 (11.43%)
Level of facility, n (%)	
Hospital	2 (5.71%)
Health Centre IV	17 (48.57%)
Health Centre III	16 (45.71%)
Health facility type, n (%)	
Public	31 (88.57%)
Private	1 (2.86%)
Private not-for-profit (PNFP)	3 (8.57%)

**Table 4:** Pre-post test scores of the participants during a CME on BEmONC/CEmONC CME: continuing medical education; BEmONC: basic emergency obstetric and newborn care; CEmONC: comprehensive emergency obstetric and newborn care

Participant number	Pre-test score (%)	Post-test score (%)
2	57	83
3	57	83
4	57	73
6	65	74
8	65	91
10	48	78
11	44	79
12	43	57
13	52	82
14	60	87
15	57	74
16	52	87
17	61	78
19	61	87
20	57	79
21	57	88
22	65	78
23	52	87
24	48	74
25	57	87
26	43	87
27	44	78
28	78	87
29	65	83
30	78	91
31	48	75
32	44	83
33	65	83
34	61	74
35	58	84
36	61	70
37	78	91
38	61	74
39	57	70
Average scores	58	80

The average pre-test score was 58% while the average post-test score was 80%. The percentage change in knowledge was 22%. The perspectives of the health providers are presented under the themes of perspectives of knowledge acquisition, perspectives of skill acquisition, and perspectives of benefits. 

Perspectives on knowledge acquisition

Participants consistently indicated an improved understanding of essential maternal and newborn health issues. They reported notable gains in understanding conditions such as eclampsia, pre-eclampsia, neonatal care, and the management of hypertension, along with practical treatment aspects, including medication administration. A midwife highlighted mastering the proper dilution of magnesium sulfate and the administration of hydralazine, illustrating the direct application of theoretical knowledge to practical service delivery.

Furthermore, the training enabled participants to strengthen their understanding of neonatal danger signs and appropriate pharmacological practices, correcting prior inaccuracies in clinical procedures. One participant reported that they now record danger signs on Manila charts and accurately administer medications, thereby improving patient safety. The training served as a refresher on previously acquired but underutilized clinical competencies, including manual vacuum aspiration (MVA) and pre-eclampsia management, reinforcing best practices and reducing uncertainty in pharmacological administration. Participants also noted that the program improved their understanding of community maternal and child health indicators, facilitating more effective monitoring and targeted intervention at the community level. This holistic perspective strengthened the connection between knowledge acquisition and improvements in overall service delivery.

Perspectives on skill acquisition

In addition to knowledge, the training was essential for the strengthening of practical skills. Numerous participants reported acquiring hands-on skills in MVA procedures, thereby reducing reliance on physicians and limiting unnecessary referrals for early-pregnancy cases. Midwives gained competencies in managing newborn asphyxia, learning to address critical conditions such as respiratory distress, arrhythmias, and seizures in neonates. A significant outcome of these skills was increased confidence in newborn resuscitation. Initially hesitant personnel reported gaining proficiency in managing ill neonates without the need for immediate referral to advanced care facilities. The CME program successfully linked theoretical knowledge with practical skills, enabling healthcare professionals to respond decisively in critical situations.

Perspectives on benefits

Participants unanimously characterized the training as highly beneficial, highlighting its role in refreshing and enhancing knowledge. Numerous individuals noted that elements of clinical care, such as the appropriate use of partographs, had been overlooked or poorly understood before the CME. The program also provided updates on emergency obstetric care (EmOC) and CEmOC skills, ensuring that even experienced practitioners are equipped with the latest best practices. The corresponding perspectives, with relevant quotes, are shown in Table 5.

**Table 5:** Healthcare providers' perpectives of knowledge, skills acquisition, and benefits RMNCAH: reproductive, maternal, newborn, child, and adolescent health; MVA: manual vacuum aspiration; CME: continuing medical education; EmOC: emergency obstetric care; CEmOC: comprehensive emergency obstetric Care

Themes	Sub-themes	Quote
Perceptions of knowledge acquisition	Improved knowledge in RMNCAH (eclampsia, pre-eclampsia, newborn care, hypertension management)	...according to what we have learned, I think we will improve in health service delivery… especially eclampsia, pre-eclampsia, and newborn babies… How to dilute magnesium sulfate and administer hydralazine... because, for me, I have never given hydralazine.” – Participant, Kabuyanda HC IV, Isingiro District
	Applying updated knowledge in newborn danger signs and drug use	“From this knowledge update… I will write on the Manila charts the danger signs in a newborn that should be looked out for… we have been using drugs, but the way we have been using them has not been good... this was addressed.” – Participant, McNell Medical Centre, Ntungamo District
	Refresher on forgotten clinical areas (MVA, pre-eclampsia)	“...in fact, it reminded me of many things which I had forgotten... MVA, pre-eclampsia... we have been fidgeting with the drugs.” – Participant, Rukunyu Hospital, Kamwenge District
	Improved understanding of community maternal and child health indicators	“It has equipped midwives with knowledge in areas of understanding community maternal and child [health] indicators, which is greatly improving the service delivery.” – Participant, Family Health Resource Center, Kiruhura District
Perceptions of skill acquisition	Acquisition of MVA procedural skills	“…..like MVA, we have not been doing MVA... we call doctors sometimes... and these doctors, when you ask them, they are also not able to do MVAs… from here I have learnt that thing, and I am sure we shall not refer any mothers below 12 weeks.” – Participant, Kabwohe HC IV, Sheema District
	Improved skills in managing newborn asphyxia	“The midwife who attended the training sessions acquired skills in helping babies directly by managing asphyxia conditions (difficulty breathing, fluctuating heart rate, weakness, and seizures or convulsions).” – Participant, Kyamuhunga HC III, Bushenyi District
	Increased confidence in newborn resuscitation	“Newborn babies scare us…, we used to refer all sick babies, but thanks to the CME of ETAT+… I have trained my colleagues here, and we are now resuscitating these babies confidently.” – Participant, Ruhija HC III, Rubanda District
Perceptions of benefits	Training is seen as beneficial in refreshing and updating knowledge	“I have really appreciated this training... it has been so beneficial... some things we had forgotten; some things we didn’t know... we have been taking partographs for granted… but now we know that the partographs can make our work easy...” – Participant, Lugazi HC IV, Rubirizi District
	Beneficial in updating EmOC/CEmOC skills	“This CME has been beneficial... I have learnt so much... I have been in the field for many years... I have taken a long time without going back to school… I have gained more skills than I had... because we had some new updates in EmOC and CEmOC...” – Participant, Kabwohe HC IV, Sheema District

## Discussion

The findings show that CME enhances knowledge acquisition, as demonstrated by the percentage improvement in pre- and post-test scores. This is consistent with studies showing that CME results in knowledge improvement [14]. Knowledge acquisition was evaluated using a pre/post survey administered during the training. Evidence indicates that a pre/post survey conducted at a single point in time after the training is not inferior to traditional pre-post surveys and produces comparable outcomes [15]. Based on the quotes, the findings also demonstrate the acquisition of practical skills among primary care providers who attended the CMEs. This is consistent with other findings reporting skill acquisition following CME among healthcare providers [15,16]. Primary care providers working in health facilities ranging from health centers to hospitals reported gaining knowledge and skills in a variety of clinical areas, indicating that CMEs are needed by care providers at all levels of the healthcare system. This is consistent with other evidence suggesting that CME programs improve clinical decision-making in primary care [17-19].

Improvement in knowledge and skills among healthcare providers becomes more pronounced because some care providers may lack prior training in specific topics, while others work under supervisors who are unfamiliar with certain areas of clinical practice and, therefore, unable to provide adequate training. In other instances, CME addresses knowledge gaps in complex and rapidly evolving medical fields [20]. Other studies have similarly supported the need for CME for these reasons [8]. Additionally, it has been reported that when CME is targeted to specific areas of clinical practice, it improves doctors’ proficiency [21]. CME has also been reported to increase care providers' confidence and motivation to perform clinical procedures as a result of enhanced knowledge. This is consistent with outcomes of CME sessions conducted elsewhere [2]. The implication is that regularly implemented CME for care providers will ensure that appropriate care standards are maintained in primary care health facilities.

Primary care providers reported that the skills acquired through CME had been applied in care provision across various health facilities. In some cases, primary care providers had previously overlooked aspects of care that were taken for granted or not considered important, while in other instances, they were hesitant to perform specific medical procedures. However, following the CME sessions, these procedures were reported to have become part of their regular practice. Through continuing education, medical practitioners can enhance their knowledge and abilities to provide patients with the best care possible while also adapting to the numerous changes in society [22].

Limitations

Our findings among primary care providers following CMEs were limited to participants from health facilities in southwest Uganda. However, the participants are representative of primary care providers in resource-constrained settings in Uganda, and their perspectives are likely similar to those in comparable settings across the country following CME sessions. Participant feedback was collected during faculty-led CME sessions, which may have introduced social desirability bias. In addition, the analysis was based on secondary qualitative data from retrospective CME activity reports rather than real-time interviews or focus groups. Although standardized templates and contemporaneous documentation were used, the researchers did not have control over the original data collection process and were unable to probe or clarify responses, potentially limiting the depth and diversity of perspectives.

## Conclusions

The findings demonstrate that university-led CME can effectively support primary care providers in resource-constrained settings to acquire knowledge and enhance their skills. Building collaborative relationships between universities and actors in the catchment health system to ensure the sustainability of CMEs is likely to have a positive effect on healthcare delivery.
